# The relation of depression with structural brain abnormalities and cognitive functioning: the Maastricht study

**DOI:** 10.1017/S0033291721000222

**Published:** 2022-11

**Authors:** Anouk F. J. Geraets, Miranda T. Schram, Jacobus F. A. Jansen, Annemarie Koster, Pieter C. Dagnelie, Marleen M. J. van Greevenbroek, Coen D. A. Stehouwer, Frans R. J. Verhey, Sebastian Köhler

**Affiliations:** 1 Alzheimer Centrum Limburg, Maastricht, the Netherlands; 2 Department of Psychiatry and Neuropsychology, Maastricht, the Netherlands; 3 Department of Internal Medicine, Maastricht, the Netherlands; 4 School for Mental Health and Neuroscience, Maastricht, the Netherlands; 5 School for Cardiovascular Diseases (CARIM), Maastricht, the Netherlands; 6 Heart and Vascular Centre, Maastricht, the Netherlands; 7 Department of Radiology, Maastricht, the Netherlands; 8 Department of Social Medicine, Maastricht University Medical Centre+ (MUMC+), Maastricht, the Netherlands; 9 Faculty of Health, Medicine & Life Sciences, Care and Public Health Research Institute (CAPHRI), Maastricht University, Maastricht, the Netherlands

**Keywords:** Cerebral small vessel disease, cognitive functioning, depression, epidemiology, population-based study

## Abstract

**Background:**

Individuals with depression often experience widespread and persistent cognitive deficits, which might be due to brain atrophy and cerebral small vessel disease (CSVD). We therefore studied the associations between depression, markers of brain atrophy and CSVD, and cognitive functioning.

**Methods:**

We used cross-sectional data from the population-based Maastricht study (*n* = 4734; mean age 59.1 ± 8.6 years, 50.2% women), which focuses on type 2 diabetes. A current episode of major depressive disorder (MDD, *n* = 151) was assessed by the Mini-International Neuropsychiatric Interview. Volumes of cerebral spinal fluid, white matter, gray matter and white matter hyperintensities, presence of lacunar infarcts and cerebral microbleeds, and total CSVD burden were assessed by 3 T magnetic resonance imaging. Multiple linear and logistic regression analyses tested the associations between MDD, brain markers and cognitive functioning in memory, information processing speed, and executive functioning & attention, and presence of cognitive impairment. Structural equation modeling was used to test mediation.

**Results:**

In fully adjusted models, MDD was associated with lower scores in information processing speed [mean difference = −0.18(−0.28;−0.08)], executive functioning & attention [mean difference = −0.13(−0.25;−0.02)], and with higher odds of cognitive impairment [odds ratio (OR) = 1.60(1.06;2.40)]. MDD was associated with CSVD in participants without type 2 diabetes [OR = 1.65(1.06;2.56)], but CSVD or other markers of brain atrophy or CSVD did not mediate the association with cognitive functioning.

**Conclusions:**

MDD is associated with more impaired information processing speed and executive functioning & attention, and overall cognitive impairment. Furthermore, MDD was associated with CSVD in participants without type 2 diabetes, but this association did not explain an impaired cognitive profile.

## Introduction

Several studies have shown that depression is strongly related to impaired cognition (Rock, Roiser, Riedel, & Blackwell, 2014). Around two-thirds of individuals with depression experience impaired cognitive functioning (Rock et al., 2014), and studies in patients with clinical depression have shown that cognitive deficits persist despite remission of depressive symptoms (Koenig, Bhalla, & Butters, 2014; Köhler, Thomas, Barnett, & O'Brien, 2010). Several prospective cohort studies suggest that individuals with depression show accelerated cognitive decline and have a two times higher risk for Alzheimer's disease (AD) and three times higher risk for vascular dementia (Mourao, Mansur, Malloy-Diniz, Castro Costa, & Diniz, 2016).

Underlying mechanisms that are involved in the etiology of dementia such as brain atrophy (Pini et al., 2016) and cerebral small vessel disease (CSVD) (O'Brien & Thomas, 2015) have also been linked to depression. Volume reductions in total gray matter (GM) (Tudorascu et al., 2014), the hippocampus (Tittmann et al., 2014), and several prefrontal regions (Tittmann et al., 2014), were reported to be associated with depression. Studies have also shown that depression is related to markers of CSVD, including white matter hyperintensities (WMH), lacunar infarcts, and cerebral microbleeds (van Agtmaal, Houben, Pouwer, Stehouwer, & Schram, 2017). Small clinical studies have demonstrated that these cerebral changes are related to cognitive functioning in memory, executive functions, and processing speed in patients with depression (Hickie et al., 2005; Köhler et al., 2010; O'Brien, Lloyd, McKeith, Gholkar, & Ferrier, 2004; Sheline et al., 2008). However, studies generally were small and confined to the more severely depressed spectrum such as inpatients, hence there is a lack of population-based cohort studies that generalize findings to the wider population with depression. Furthermore, most studies only corrected for a limited number of confounders (Hickie et al., 2005; Köhler et al., 2010; O'Brien et al., 2004; Sheline et al., 2008) and associations found could be spurious; studies mostly included a limited number of brain markers (Hickie et al., 2005; O'Brien et al., 2004), which might have resulted in less sensitivity; and studies were often performed in elderly populations (>59 years) (Köhler et al., 2010; O'Brien et al., 2004; Sheline et al., 2008), while the development of structural brain damage may already start at middle age (Jernigan et al., 2001).

Therefore, the aim of this study is to investigate the association between depression, brain volume (white matter (WM), GM), markers of generalized atrophy (cerebrospinal fluid (CSF) volume) and CSVD (WMH, lacunar infarcts, and cerebral microbleeds), cognitive impairment and functioning (memory, information processing speed, and executive function & attention). In addition, we assess whether markers of brain atrophy and CSVD mediate the association between depression and cognition. Previous studies that related structural brain abnormalities to depression and cognition mainly included elderly populations, and sex differences are found in both MDD (WHO, 2017) and structural brain abnormalities (Ruigrok et al., 2014). Furthermore, it has been shown that type 2 diabetes mellitus (T2DM) is associated with MDD (Roy & Lloyd, 2012), cognitive functioning (Reijmer, van den Berg, Ruis, Jaap Kappelle, & Biessels, 2010), and structural brain abnormalities (Vergoossen, Jansen, Backes, & Schram, 2020). Because of this, we tested whether associations differed according to sex, age, and T2DM status in a cohort enriched for T2DM. We hypothesized that depression is associated with worse cognitive functioning and that these associations are partly explained by markers of brain atrophy and CSVD. In addition, we expected that the associations are similar between the individuals with and without T2DM.

## Method

### Study population and design

**Fig. 1.**
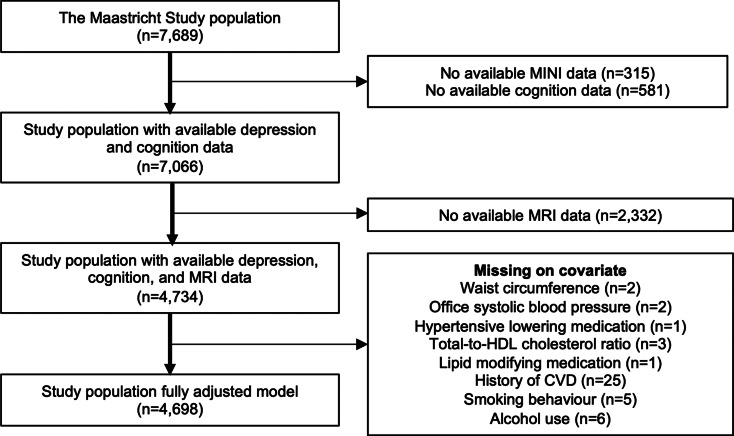
Flowchart of the study population. MINI indicates Mini-International Neuropsychiatric Interview; MRI, magnetic resonance imaging; HDL, high-density lipoprotein; CVD, cardiovascular disease.

We used cross-sectional data from the Maastricht study, an observational population-based cohort study. The rationale and methodology have been described previously (Schram et al., 2014). In brief, the study focuses on the etiology, pathophysiology, complications, and comorbidities of T2DM, heart disease, and other chronic conditions, and is characterized by an extensive phenotyping approach. Eligible for participation were all individuals aged between 40 and 75 years and living in the southern part of the Netherlands. Participants were recruited through mass media campaigns, the municipal registries, and the regional Diabetes Patient Registry via mailings. Recruitment was stratified according to known T2DM status, with an oversampling of individuals with T2DM, for reasons of efficiency. Fig. 1 shows the flowchart of the study population. Baseline surveys were completed between November 2010 and January 2018. From the initial 7689 participants, depression and cognitive functioning data was available in *n* = 7066 participants. Magnetic resonance imaging (MRI) measurements were implemented from December 2013 onwards until February 2017 and were available in 4734 participants. We performed complete case analyses in which 4734 participants were included in the key analyses and 4698 participants in the fully adjusted analyses. All participants gave written informed consent.

### Major depressive disorder

Current and lifetime episodes of major depressive disorder (MDD) were assessed by the Mini-International Neuropsychiatric Interview (MINI) (Sheehan et al., 1998). The MINI is a short diagnostic structured interview used to assess the presence of MDD in the preceding 2 weeks, in line with the DSM-IV (Diagnostic and Statistical Manual of Mental Disorders, Fourth Edition). MDD is diagnosed if participants had (1) one core symptom (i.e. depressed mood or loss of interest) and at least four other symptoms of depression (i.e. significant weight change of change in appetite, insomnia or hypersomnia, psychomotor agitation or retardation, fatigue or loss of energy, guilt or worthlessness, diminished ability to think or concentrate or indecisiveness, and suicidal thoughts or plans), or (2) two core symptoms and at least three other symptoms, for a period of more than 2 weeks. The MINI was conducted by trained staff members.

### Cognitive performance

Cognitive performance was assessed by a concise (30-min) neuropsychological test battery (Schram et al., 2014). For conceptual clarity, test scores were standardized and divided into three cognitive domains (memory function, information processing speed, and executive function & attention). Briefly, memory function was evaluated using the Verbal Learning Test (Van Der Elst, Van Boxtel, Van Breukelen, & Jolles, 2005), and a memory domain score was derived by calculating the average of total immediate and delayed recall standardized scores. An information processing speed domain score was derived from standardized scores of the Stroop Color-Word Test Parts I and II (Van der Elst, Van Boxtel, Van Breukelen, & Jolles, 2006c), the Concept Shifting Test Parts A and B (Van der Elst, Van Boxtel, Van Breukelen, & Jolles, 2006a), and the Letter-Digit Substitution Test (Van der Elst, van Boxtel, van Breukelen, & Jolles, 2006b). The executive function & attention domain score was calculated the average of the Stroop Color-Word Test Part III and the Concept Shifting Test Part C. If necessary, individual test scores were log-transformed to reduce the skewness of distributions and/or inverted so that higher scores indicated better cognitive performance. In addition, participants were categorized as cognitively impaired (yes/no) based on a regression-based normalization procedure per test that predicted expected scores for each individual given their age, sex, and level of education from a published normative sample (Van Der Elst et al., 2005, 2006*a*, *b*, *c*). The difference between observed and expected scores and their standard deviation were used to calculate *z*-scores, which were then averaged per domain and re-standardized. Individuals performing less than −1.5 standard deviations below their norm-based expected score in any domain were categorized as having a significant cognitive impairment (CogImp).

### Brain magnetic resonance imaging

Brain MRI was performed on a 3 T MRI scanner (MAGNETOM Prismafit Syngo MR D13D; Siemens Healthcare, Erlangen, Germany) by use of a 64-element head coil for parallel imaging, as previously described (Li et al., 2020). The MRI protocol consists of a 3D T_1_-weighted sequence (TR/TE/TI2300/2.98/900 ms, 1.00 mm cubic voxel, 176 continuous slices, matrix size of 240 × 250 and reconstructed matrix size of 512 × 512), a T_2_-weighted fluid-attenuated inversion recovery (TR/TE/TI 5000/394/1800 ms, 0.98 × 0.98 × 1.26 mm^3^ acquisition voxel and 0.49 × 0.49 × 1.00 mm^3^ reconstructed voxel, 176 continuous slices, acquisition matrix size of 250 × 250 and reconstructed matrix size of 512 × 512), and a gradient recalled echo pulse sequence with susceptibility-weighted imaging. The protocols for MRI acquisition and analysis are in line with current STRIVE V2 imaging standards (Wardlaw et al., 2013).

T_1_ images and T_2_-weighted fluid-attenuated inversion recovery images were analysed by use of an ISO-13485:2012-certified, automated method (De Boer et al., 2009; Vrooman et al., 2007). T_1_-weighted images were segmented into WM, GM, and CSF, volumes (1 voxel = 1.00 mm^3^ = 0.001 ml). Intracranial volume was calculated as the sum of WM (including WMH volume), GM, and CSF volume. Volumes of WM, GM, CSF, and intracranial volume were standardized.

T_1_ images and T_2_-weighted fluid-attenuated inversion recovery images were used to quantify deep, periventricular, and total WMH volume. Periventricular WMH were automatically defined as WMH <3 mm, and deep cortical WMH as WMH ⩾ 3 mm from the CSF volume. WMH volumes were log-transformed and standardized. In addition, WMH were visually rated with the Fazekas scale (Fazekas, Chawluk, Alavi, Hurtig, & Zimmerman, 1987). The location and the number of lacunar infarcts are manually rated on T2 and fluid-attenuated inversion recovery images and defined as focal lesions of ⩾3 mm and <15 mm in size with a similar signal intensity as CSF on all sequences and a hyperintense rim (Wardlaw et al., 2013). The location and the number of cerebral microbleeds are manually rated on 3D T2* gradient recalled echo-weighted imaging with susceptibility-weighted imaging by use of the Microbleed Anatomical Rating Scale (Gregoire et al., 2009) and defined as focal lesions of ⩾2 mm and ⩽10 mm in size with a hypointense signal on T2* gradient recalled echo and susceptibility-weighted images (Wardlaw et al., 2013). Lacunar infarcts and cerebral microbleeds were rated manually by three neuroradiologists. The intraclass correlation coefficient for the three raters based on 50 randomly selected scans was 0.84 (0.74; 0.91) and 0.83 (0.72; 0.90) for the presence of lacunar infarcts and cerebral microbleeds, respectively. Presence of CSVD was defined as (1) presence of lacunar infarct, (2) presence of cerebral microbleeds, and/or (3) Fazekas score ⩾2.

Because MRI assessment did not always take place at the same time as the baseline assessment for logistic reasons, we adjusted for MRI lag time, i.e. the time between baseline assessment and MRI assessment.

### General characteristics and covariates

General characteristics and covariates were measured as described elsewhere (Schram et al., 2014). Educational level (low, intermediate, high), partner status (partner/no partner), history of cardiovascular diseases (CVD), smoking status (never, current, former), alcohol consumption (none, low, high), physical activity, and Mediterranean diet score were assessed by questionnaires (Schram et al., 2014). We measured height, weight, waist circumference, blood pressure (measured in office and via ambulatory 24-h blood pressure monitoring at home (WatchBP03; Microlife AG, Widnau, Switzerland)), serum creatinine, 24-h urinary albumin excretion (twice), and plasma lipid profile as described elsewhere (Schram et al., 2014). Estimated glomerular filtration rate (eGFR; in ml/min/1.73 m^2^) was calculated with the Chronic Kidney Disease Epidemiology Collaboration equation based on both serum creatinine and serum cystatin C (Martens et al., 2016). Medication use was assessed in a medication interview where the generic name, dose, and frequency were registered. To determine T2DM status, all participants (except those who used insulin) underwent an oral glucose tolerance test (OGTT) after an overnight fast as previously described (Schram et al., 2014). T2DM was defined according to the World Health Organization (2006) criteria (fasting blood glucose ⩾7.0 mmol/l or a 2-h post-load blood glucose ⩾11.1 mmol/l or used oral glucose-lowering medication or insulin) (WHO, 2006).

### Statistical analyses

All statistical analyses were performed by use of the Statistical Package for Social Sciences (version 25.0; IBM, Chicago, IL, USA), Stata (version 13; StataCorp LLC, College Station, TX, USA), and Mplus (version 8.4; Muthén & Muthén, Los Angeles, CA, USA). To assess associations of MDD with cognitive functioning and MRI markers, we used multiple linear and logistic regression analyses reporting the unstandardized regression coefficient B and the odds ratio (OR), respectively, with their 95% confidence interval (CI). The associations were adjusted for potential confounders: model 1 included MDD (for cognitive outcome measures) or MDD + MRI time lag (for categorical MRI outcome measures) or MDD + MRI time lag + intracranial volume (for MRI volume-outcome measures); model 2 additionally adjusted for age, sex and educational level; model 3 additionally adjusted for T2DM (because of oversampling); model 4 additionally adjusted for waist circumference, office systolic blood pressure, hypertensive medication, total-to-high-density lipoprotein cholesterol ratio, lipid-modifying medication, and history of CVD, and model 5 additionally adjusted for smoking behavior and alcohol use. Model 3 was considered the main model. Models 4 and 5 were tested separately as covariates might well be on the causal pathway with potential overcorrection (Schisterman, Cole, & Platt, 2009). We also tested the interactions of MDD with age, sex, and T2DM on cognitive functioning and the brain markers. These interactions were first tested in main model 3, and later in models 4 and 5 that may include variables that are on the causal pathway. To test for mediation of the association between depression and cognition by brain markers, structural equation modeling was used to decompose total effects of depression into direct and indirect effects using maximum likelihood estimation for continuous and mean and variance corrected weighted least squares robust estimation for categorical dependent variables. Also, these analyses were performed in main model 3, as models 4 and 5 may include variables that might be on the causal pathway. A two-sided *p* value <0.05 was considered statistically significant.

## Results

### General characteristics of the study population

Table 1 shows the general characteristics of the study population (*n* = 4734), stratified for MDD status. Participants had a mean age of 59.1 ± 8.6 years and 50.2% were women. Participants with MDD were less educated and had a worse cardiometabolic risk profile compared to participants without MDD. Participants without MINI and cognition data (*n* = 623) were significantly older, were more often men, had a lower level of education, a worse cardiometabolic risk profile, more often T2DM, lower cognitive functioning scores, smaller brain volumes, and larger volumes of WMH than participants with MINI and cognition data. Participants without MRI data (*n* = 2332) were significantly older, had a lower level of education, a worse cardiometabolic risk profile, more often T2DM, a higher depressive symptoms score, and lower cognitive functioning scores as compared to participants with MRI data (data not shown).

**Table 1.** General characteristics study population according to depression status

Characteristic	No major depressive disorder (*n* = 4583)	Major depressive disorder (*n* = 151)	*p* value
**Demographics**			
Age (years)	59.2 ± 8.6	58.3 ± 8.4	0.233
Sex, *n* (% female)	2,30 (50.2)	75 (49.7)	0.934
Educational level, low/medium/high, *n* (%)	1397/1308/1878 (30.5/28.5/41.0)	71/48/32 (47.0/31.8/21.2)	<0.001
Partner status, *n* (% partner)	3901 (85.2)	107 (70.9)	<0.001
**Depression**			
Depressive symptoms (PHQ-9 score)	2 [0–4]	9 [6–15]	<0.001
Anti-depressive medication, *n* (%)	273 (6.0)	43 (28.5)	<0.001
Sleeping medication, *n* (%)	93 (2.0)	9 (6.0)	0.005
Anxiolytic medication, *n* (%)	90 (2.0)	16 (10.6)	<0.001
Anti-psychotic medication, *n* (%)	22 (0.5)	8 (5.3)	<0.001
**Cardiovascular risk factors**			
Body mass index (kg/m^2^)	26.4 ± 4.1	28.3 ± 5.1	<0.001
Waist circumference (cm)	93.5 ± 12.6	98.2 ± 15.2	<0.001
Office systolic BP (mmHg)	132.7 ± 17.3	134.6 ± 17.2	0.194
Office diastolic BP (mmHg)	75.4 ± 9.7	77.1 ± 10.4	0.042
Antihypertensive medication, *n* (%)	1465 (32.0)	64 (42.4)	0.010
Hypertension, *n* (%)	2230 (48.7)	90 (59.6)	0.010
Total/high density cholesterol ratio	3.6 ± 1.2	3.8 ± 1.3	0.012
Triglycerides (mmol/l)	1.4 ± 0.8	1.5 ± 0.8	0.005
Lipid-modifying medication, *n* (%)	1225 (26.7)	49 (32.5)	0.135
eGFR (ml/min/1.73 m^2^)	89.1 ± 14.1	89.7 ± 14.8	0.714
Albuminuria, normal/micro/macro, *n* (%)	1716/111/6 (93.6/6.1/0.3)	55/9/0 (85.9/14.1/0.0)	0.016
History of CVD, *n* (%)	549 (12.0)	25 (16.7)	0.098
Type 2 diabetes mellitus, *n* (%)	864 (18.9)	49 (32.5)	<0.001
Diabetes medication (all types), *n* (%)	630 (13.7)	37 (24.5)	<0.001
HbA1c (mmol/mol)	38.5 ± 8.4	42.2 ± 12.8	<0.001
**Life style factors**			
Smoking, never/former/current, *n* (%)	1814/2232/534 (39.6/48.7/11.7)	54/66/29 (36.2/44.3/19.5)	0.078
Alcohol use, none/low/high, *n* (%)	750/2682/1146 16.4/58.6/25.0	47/79/24 31.3/52.7/16.0	<0.001
Physical activity (h/week)	14.2 ± 8.0	12.8 ± 8.3	0.047
Mediterranean diet score	4.5 ± 1.7	4.1 ± 1.8	0.001
**Cognitive functioning**			
Memory score	0.09 ± 0.93	−0.06 ± 0.89	0.061
Information processing speed score	0.09 ± 0.75	−0.18 ± 0.82	<0.001
Executive functioning & attention score	0.09 ± 0.78	−0.15 ± 0.86	<0.001
Cognitive impairment, *n* (%)	648 (14.1)	35 (23.2)	0.003
**Markers of brain atrophy and CSVD**			
Cerebrospinal fluid (ml)	252.2 ± 47.7	244.8 ± 48.7	0.062
White matter (ml)	476.2 ± 58.4	464.4 ± 63.8	0.015
Gray matter (ml)	662.5 ± 60.6	649.6 ± 68.6	0.010
Intracranial volume (ml)	1391.8 ± 133.4	1359.7 ± 147.4	0.004
WMH (ml)	0.21 [0.06–0.66]	0.25 [0.08–0.72]	0.161
Deep cortical WMH (ml)	0.04 [0.01–0.19]	0.04 [0.01–0.19]	0.559
Periventricular WMH (ml)	0.14 [0.04–0.46]	0.16 [0.05–0.52]	0.120
Fazekas score ⩾2, *n* (%)	1054 (23.0)	38 (25.2)	0.556
Cerebral lacunar infarct, *n* (%)	197 (4.3)	8 (5.3)	0.539
Cerebral microbleeds, *n* (%)	458 (10.0)	15 (9.9)	1.000
CSVD, *n* (%)	1437 (31.4)	52 (34.4)	0.424
MRI lag time (years)	0.70 [0.02–1.30]	0.80 [0.23–2.08]	0.482

Data are presented as means ± standard deviation (s.d.), number (%) or median [interquartile range], and evaluated using independent *t* tests, Mann–Whitney *U* tests or χ^2^ tests. PHQ-9 indicates 9 item Patient Health Questionnaire; BP, blood pressure; HDL, high-density lipoprotein; eGFR, estimated glomerular filtration rate; CVD, cardiovascular disease; CSVD, cerebral small vessel disease; HbA1c, glycated hemoglobin A1c; WMH, white matter hyperintensities.

### Association between MDD and cognitive functioning

Table 2 shows the association between MDD and cognitive functioning. MDD was associated with higher odds of CogImp [OR = 1.60(1.06;2.40)] after full adjustment for demographic, cardiovascular, and lifestyle risk factors (model 5). Furthermore, MDD was associated with lower scores in information processing speed [*B* = −0.18(−0.28;−0.08)] and executive functioning & attention [*B* = −0.13(−0.25;−0.02)], but not with memory [*B* = −0.02(−0.15;0.11)] in model 5. We did not find interactions of MDD with age, sex, or T2DM on CogImp or cognitive domain scores (online Supplementary Table 1 S1).

**Table 2.** Associations of major depressive disorder with different domains of cognitive functioning

	Memory score		Information processing speed score		Executive functioning & attention score		Cognitive impairment (yes/no)	
Model	Mean difference (95% CI)	*p* value	Mean difference (95% CI)	*p* value	Mean difference (95% CI)	*p* value	OR (95% CI)	*p* value
Model 1	−0.15 (−0.30;0.01)	0.061	−0.27 (−0.40;−0.15)	<0.001	−0.23 (−0.36;−0.11)	<0.001	1.83 (1.24;2.70)	0.002
Model 2	−0.08 (−0.21;0.06)	0.257	−0.22 (−0.33;−0.12)	<0.001	−0.18 (−0.29;−0.06)	0.002	1.89 (1.28;2.79)	0.001
Model 3	−0.06 (−0.19;0.07)	0.373	−0.21 (−0.31;−0.10)	<0.001	−0.17 (−0.28;−0.05)	0.005	1.79 (1.21;2.66)	0.004
Model 4	−0.05 (−0.18;0.08)	0.447	−0.20 (−0.30;−0.10)	<0.001	−0.16 (−0.27;−0.04)	0.008	1.74 (1.17;2.59)	0.007
Model 5	−0.02 (−0.15;0.11)	0.735	−0.18 (−0.28;−0.08)	0.001	−0.13 (−0.25;−0.02)	0.026	1.60 (1.06;2.40)	0.024

*n* = 4734. Major depressive disorder cases *n* = 151. Regression results are presented as mean difference or odds ratio (OR) with 95% confidence intervals (95% CI). CI indicates confidence interval; OR, odds ratio.

**Model 1:** crude.

**Model 2:** adjusted for age, sex, and educational level.

**Model 3:** additionally adjusted for type 2 diabetes mellitus.

**Model 4:** additionally adjusted for waist circumference, office systolic blood pressure, hypertensive medication, total/high-density cholesterol ratio, lipid-modifying medication, and history of cardiovascular disease (*n* = 4701).

**Model 5:** additionally adjusted for smoking behavior and alcohol use (*n* = 4698).

### Association between MDD and markers of brain atrophy and CSVD

Table 3 shows the associations between MDD and markers of brain atrophy and CSVD. MDD was not associated with WM volume [*B* = 0.00(−0.08;0.07)], GM volume [*B* = −0.01(−0.09;0.06)], or CSF volume [*B* = 0.02(−0.08;0.12)], after adjustment for demographic risk factors (model 2). MDD was associated with larger WMH volume [*B* = 0.16(0.02 to 0.30)] after adjustment for demographic risk factors (model 2), but not after additional adjustment for T2DM in model 3 [*B* = 0.14(0.00;0.28)]. However, the association between MDD and periventricular WMH volume remained significant after additional adjustment for T2DM in model 3 [*B* = 0.15(0.01;0.29)], and became non-significant after additional adjustment for potentially mediating cardiovascular risk factors in model 4 [*B* = 0.14(0.00;0.28)]. MDD was not associated with deep cortical WMH volume [*B* = 0.11(−0.03;0.26)] or presence of CSVD [OR = 1.32(0.91;1.91)] after adjustment for demographic risk factors in model 2.

**Table 3.** Associations of major depressive disorder with markers of brain atrophy and cerebral small vessel disease

	CSF volume (per 1 s.d.)	WM volume (per 1 s.d.)	GM volume (per 1 s.d.)	WMH volume (per 1 s.d.)	DWMH volume (per 1 s.d.)	PWMH volume (per 1 s.d.)	CSVD (yes/no)
Model	Mean difference (95% CI)	Mean difference (95% CI)	Mean difference (95% CI)	Mean difference (95% CI)	Mean difference (95% CI)	Mean difference (95% CI)	OR (95% CI)
Model 1	−0.01 (−0.14;0.12)	0.01 (−0.07;0.09)	−0.01 (−0.09;0.08)	0.11 (−0.05;0.27)	0.06 (−0.10;0.22)	0.13(−0.04;0.29)	1.13(0.80;1.59)
Model 2	0.02 (−0.08;0.12)	0.00 (−0.08;0.07)	−0.01 (−0.09;0.06)	0.16 (0.02;0.30)	0.11 (−0.03;0.26)	0.18(0.04;0.32)	1.27(0.88;1.83)
Model 3	−0.01 (−0.11;0.10)	0.01 (−0.07;0.08)	0.00 (−0.07;0.07)	0.14 (−0.00;0.28)	0.09 (−0.06;0.23)	0.15(0.01;0.29)	1.24(0.86;1.79)
Model 4	−0.01 (−0.11;0.09)	0.00 (−0.07;0.08)	0.00 (−0.07;0.08)	0.12 (−0.02;0.26)	0.08 (−0.07;0.22)	0.14(0.00;0.28)	1.22(0.84;1.76)
Model 5	−0.01 (−0.11;0.09)	−0.02 (−0.14;0.10)	0.01 (−0.07;0.08)	0.12 (−0.02;0.26)	0.07 (−0.08;0.21)	0.13(−0.01;0.27)	1.23(0.85;1.79)

*n* = 4734. Major depressive disorder cases *n* = 151. Regression results are presented as mean difference or odds ratio (OR) with 95% confidence intervals (95% CI). CSF indicates cerebrospinal fluid; WM, white matter; GM, gray matter; s.d., standard deviation; WMH, white matter hyperintensity; DWMH, deep cortical white matter hyperintensities; PWMH, periventricular white matter hyperintensities; CSVD, cerebral small vessel disease.

**Model 1:** adjusted for intracranial volume (except CSVD composite score) and MRI lag time.

**Model 2:** additionally adjusted for age, sex, and educational level.

**Model 3:** additionally adjusted for type 2 diabetes mellitus.

**Model 4:** additionally adjusted for waist circumference, office systolic blood pressure, hypertensive medication, total/high-density cholesterol ratio, lipid-modifying medication, and history of cardiovascular disease (*n* = 4701).

**Model 5:** additionally adjusted for smoking behavior and alcohol use (*n* = 4698).

Next, we tested whether the above associations differ by age, sex and T2DM in model 3 (online Supplementary Table S2). We found an interaction of MDD with age for WM volume in model 3 (*p* = 0.029), suggesting MDD is associated with a larger WM volume in older participants only. The interaction attenuated and became non-significant in model 4 (*p* = 0.082), which could be attributed to differences in blood pressure between young and old individuals with MDD. Next, an interaction of MDD with age for GM existed in model 3 (*p* = 0.013) which remained statistically significant in model 5 (*p* = 0.023). Results of stratified analyses were suggestive for an association between MDD and lower GM volume in older participants [⩾65 years, model 3: *B* = −0.12(−0.29;0.06)], while no association was observed in younger participants [<65 years, model 3: *B* = 0.03(−0.05;0.10)]. However, results in the older age group were statistically non-significant. Finally, we found an interaction of MDD with T2DM on CSVD score in model 3 (*p* = 0.026) which remained statistically significant in the fully adjusted model 5 (*p* = 0.023). Stratified analyses of model 5 showed that MDD was associated with the presence of CSVD in participants without T2DM [OR = 1.65(1.06;2.56)], but not in participants with T2DM [OR = 0.68(0.34;1.38)]. In the former, MDD was also significantly associated with WHM volume [*B* = 0.17(0.01;0.34)] and periventricular WMH volume [*B* = 0.18(0.01;0.35)] in model 3, but not after adjustment for cardiovascular and lifestyle factors.

### Mediation analyses of the association between MDD, CSVD, and cognition

In the above results, we only found an association between MDD and markers of CSVD in the subpopulation without T2DM. Therefore, only mediation analyses in the subpopulation without T2DM are shown in Table 4. Mediation analyses of the total study population are shown in online Supplementary Table S4. There was a modest indirect effect for periventricular WMH [model 2: *B* = −0.02(−0.03;−0.00)] on memory, in which 20% of the total effect of MDD on memory score could be attributed to its relation with periventricular WMH. Since the total effect itself was not significant itself, this is a marginal association. There were no mediation effects for other cognitive outcomes (Table 4).

**Table 4.** Decomposed associations of major depressive disorder with different domains of cognitive functioning in the subpopulation without type 2 diabetes

	Memory score	Information processing speed score	Executive functioning & attention score	Cognitive impairment (yes/no)
Model	Mean difference (95% CI)	Mean difference (95% CI)	Mean difference (95% CI)	OR (95% CI)
**WMH**				
Direct	−0.08 (−0.23;0.07)	−0.18 (−0.30;−0.06)*	−0.12 (−0.25;0.02)	1.19 (0.89;1.61)
Indirect	−0.02 (−0.03;0.00)	−0.01 (−0.02;0.00)	−0.01 (−0.02;0.00)	1.02 (1.00;1.05)
Total	−0.10 (−0.25;0.06)	−0.18 (−0.31;−0.06)*	−0.13 (−0.26;0.01)	1.22 (0.91;1.64)
**DWMH**				
Direct	−0.08(−0.24;0.07)	−0.18 (−0.30;−0.06)*	−0.12 (−0.26;0.01)	1.20 (0.89;1.61)
Indirect	−0.01(−0.03;0.00)	−0.01 (−0.02;0.00)	−0.01 (−0.01;0.00)	1.02 (1.00;1.05)
Total	−0.10(−0.25;0.06)	−0.18 (−0.31;−0.06)*	−0.13 (−0.26;0.01)	1.22 (0.91;1.64)
**PWMH**				
Direct	−0.08 (−0.23;0.07)	−0.18 (−0.30;−0.06)*	−0.12 (−0.25;0.02)	1.20 (0.89;1.62)
Indirect	−0.02 (−0.03;−0.00)*	−0.01 (−0.01;0.00)	−0.01 (−0.02;0.00)	1.02 (1.00;1.04)
Total	−0.10 (−0.25;0.06)	−0.18 (−0.31;−0.06)*	−0.13 (−0.26;0.01)	1.22 (0.91;1.64)
**CSVD**				
Direct	−0.07 (−0.25;0.10)	−0.19 (−0.31;−0.07)*	−0.14 (−0.28;−0.00)*	1.21 (0.91;1.62)
Indirect	−0.01 (−0.03;0.00)	−0.01 (−0.02;0.00)	−0.00 (−0.01;0.01)	1.03 (0.99;1.06)
Total	−0.08 (−0.26;0.09)	−0.20 (−0.32;−0.08)*	−0.15 (−0.28;−0.01)*	1.25 (0.93;1.67)

Regression results are decomposed in direct, indirect and total effects and presented as mean difference or odds ratio (OR) with 95% confidence intervals (95% CI). CI indicates confidence interval; OR, odds ratio; MDD, major depressive; WMH, white matter hyperintensities; DWMH, deep cortical white matter hyperintensities; PWMH, periventricular white matter hyperintensities; CSVD, cerebral small vessel disease. Model 2 is adjusted for adjusted for intracranial volume (except CSVD composite score), MRI lag time, age, sex, educational level. *n* = 3821, MDD cases *n* = 102. *p < 0.05 (two-sided).

### Additional analyses

Results of additional analyses are shown in online Supplementary Tables S5–7. Since more participants had data on cognitive functioning than on MRI, we inspected potential selection bias by including only those with available MRI. Overall, associations between MDD and cognitive functioning were attenuated in the sample with available MRI compared to the full sample analyses (online Supplementary Table S5). Next, we performed several sensitivity analyses. First, to reduce potential misclassification of depression, we (1) additionally adjusted for anti-depressant medication, (2) exclude participants who used anti-depressant medication from the control group, (3) exclude participants with moderate to high depressive symptoms (PHQ-9 score ⩾10) from the control group, and (4) exclude participants without MDD at baseline who did report a lifetime diagnosis of MDD from the control group. Second, we additionally adjusted for physical activity, Mediterranean diet score, and eGFR, which were missing in more participants and therefore not included in the main analyses. Third, we replaced office systolic blood pressure for 24 h systolic blood pressure, waist circumference for BMI, and total-to-HDL cholesterol ratio for triglycerides. All these adjustments did not materially change our results (online Supplementary Table S6). Previous studies that investigated structural brain abnormalities often focused on late-life depression above the age of 60 years (Blazer, 2003). To investigate whether associations between MDD and markers of brain atrophy and CSVD become stronger with age, we tested these associations in several age categories (online Supplementary Table S7). This showed that associations between MDD and markers of brain atrophy and CSVD indeed became stronger with increasing age.

## Discussion

In this population-based imaging study, MDD was associated with higher odds of cognitive impairment, and more impaired performance in information processing speed and executive functioning & attention, but not memory. These associations were independent of demographic, cardiovascular, and lifestyle-related risk factors, and were similar in women and men, and in participants with and without T2DM. Contrary to our expectations, MDD was not associated with markers of brain atrophy or CSVD in the total study population. However, MDD was associated with CSVD in participants without T2DM, but this relation could not explain the associations between MDD and cognitive functioning.

Our finding that MDD is associated with generalized cognitive impairment corroborates previous evidence of a relation between depression and cognitive deficits (Rock et al., 2014), and are in line with studies that show that these cognitive deficits are often persistent (Köhler et al., 2010) and can increase over time (Mourao et al., 2016). Cognitive impairment persists in 45% of patients even after remission of MDD (Thomas & O'Brien, 2008).

We found participants with MDD and no T2DM had more evidence for CSVD, which mirrors previous findings (van Agtmaal et al., 2017). The association between MDD and total WMH volume was statistically non-significant, while the association between MDD and periventricular WMH volume was statistically significant, after adjustment for demographic risk factors and T2DM. However, the difference in the strength of the association between MDD with respectively total WMH volume and periventricular WMH volume is <7%. In addition, the CIs of these associations are almost identical. The non-significant association with total WMH can be explained by the absence of an association between MDD and deep cortical WMH volume in our study population, which diluted the association between MDD and total WMH volume. Although some studies differentiate between periventricular and deep cortical WMH volume, results of a meta-analysis suggested that this categorical distinction may be arbitrary (DeCarli, Fletcher, Ramey, Harvey, & Jagust, 2005).

Others also found an association between depression and brain atrophy (Tudorascu et al., 2014), but studies into whole-brain GM volume have shown mixed results (Tittmann et al., 2014). More consistent evidence has been found for volume reductions in specific brain areas such as the hippocampus, prefrontal cortex, insula, putamen, amygdala, and anterior cingulate-, prefrontal, and temporal cortex (Tittmann et al., 2014). Moreover, it has been shown that the effect of depression on frontal and temporal GM volume reductions increases with age (Dotson, Davatzikos, Kraut, & Resnick, 2009), for which we found tentative support as the interaction between MDD and age on GM suggested that MDD is more strongly related to lower GM volumes in older age (Tudorascu et al., 2014). The reduced statistical power in the stratified analyses can explain the non-significant association between MDD and lower GM volume in the older subgroup. It may be that our study population was still too young (mean age 59.1 ± 8.6 years) with limited variation in brain volumes to find an association between MDD and markers of brain atrophy, and consequently, a mediation of brain atrophy on the association between MDD and cognitive impairment as brain volume loss might have been subtle. Our finding that MDD is stronger associated with information processing speed and executive functioning & attention as compared to memory is more in line with an underlying vascular mechanism than a neurodegenerative mechanism involving the hippocampus (Koenig et al., 2014).

As said, depressed participants without T2DM more often had signs of CSVD. In addition, total WMH and periventricular WHM load were higher. This was partly explained by their worse general vascular health profile since associations with WMH and periventricular WMH became non-significant after adjustment for cardiovascular factors. Previous studies that adjusted for factors such as hypertension also reported modest associations (van Agtmaal et al., 2017). The relatively young age and the general absence of large confluent WHM (Fazekas score ⩾3, *n* = 37) in our sample may explain the subtler associations. The use of the categorical CSVD variable might have allowed a sharper contrast between those with high and low CSVD burden. As a consequence, associations between MDD and CSVD were comparable with a recent meta-analysis on the presence of significant WMH in MDD (van Agtmaal et al., 2017).

Despite the above associations, neither CSVD nor markers of brain atrophy explained the worse cognitive profile of individuals with MDD in the total sample or among those without T2DM. It is possible that markers of CSVD and cognitive impairment are only related within a subgroup of depression (so-called vascular depression) (Sneed, Rindskopf, Steffens, Krishnan, & Roose, 2008), but such associations might be diluted at the population-average level. Contrary to our expectation, we did not observe an association between MDD and markers of brain atrophy or CSVD in individuals with T2DM. This might be because vascular burden in T2DM is already high. Although it has been shown that comorbid depression in T2DM is related to the development of macro- and micro-vascular complications (Nouwen et al., 2019), the presence of MDD might add little to the variation in the observed association with CSVD (van Agtmaal et al., 2018).

Several mechanisms have been suggested to explain the higher risk of cognitive impairment in individuals with MDD. This includes neurobiological effects of depression such as chronically increased cortisol levels and glutamatergic neurotoxicity (Odaka, Adachi, & Numakawa, 2017), and decreased in heartrate variability and an increase in platelet activation and pro-inflammatory factors (Carney, Freedland, & Veith, 2005). On the other hand, brain atrophy or ischemic damage in affect regulating centers (fronto-subcortical loop) might be a common etiological factor for both MDD and cognitive impairment. In that case, brain markers could be seen as a classical confounder for the association between MDD and cognitive impairment, though reported direct effects of MDD on cognition remained significant in models including the MRI markers. Next, lower GM volumes might be a marker of impending dementia (Thompson et al., 2003) and the association be due to reverse causation. The stronger association in older age groups between MDD and GM seems in line with this, though we would have expected to find associations with intracranial CSF volumes, as a proxy of generalized brain atrophy, in particular. Finally, cognitive impairments might be a state effect of a current depressive episode. Yet, several studies suggest impairments are highly persistent or worsening in depression (Thomas & O'Brien, 2008). Importantly, the above explanations are not mutually exclusive and different pathways might act in different individuals with accumulation and interaction of their effects.

Strengths of our study include its large sample size and population-based design; the oversampling of individuals with T2DM which provides insights of the investigated associations in this population with a high burden of depression (Holt, De Groot, & Golden, 2014), cognitive impairment and brain changes; the comparable prevalence rate of depression to other population-based studies; the use of the MINI diagnostic interview to assess MDD; the extensive assessment cognitive functioning by means of a comprehensive neuropsychological test battery with available norm scores to define cognitive impairment; inclusion of a broad range of potential confounders; and the performance of several sensitivity analyses to test the robustness of findings.

This study also has some limitations. First, the data were cross-sectional. Therefore, we cannot exclude reverse causality. Second, the study population was relatively young, with an upper age range of 75 years, and despite the oversampling of T2DM relatively healthy, which may have led to an underestimation of the associations with brain atrophy and CSVD. These effects appear to be more pronounced in older adults, as most studies who found these effects included participants above the age of 65 years. In addition, functional MRI research has linked MDD to abnormal functioning of cognition-related brain networks in individuals with an MDD onset below the age of 50 years (Rayner, Jackson, & Wilson, 2016). Research into more subtle markers of brain changes, like brain connectivity or functional measures, is therefore recommended for younger populations. Third, we adjusted for a range of potential confounders, but due to the cross-sectional assessment, we could not differentiate between cause and effect. Cardiovascular and lifestyle factors might very well be on the causal pathway from depression to brain changes, especially CSVD. This overadjustment might explain the loss of significant associations in models 4 and 5 in the analyses with WMH and periventricular WMH, and therefore we kept these factors in separate models (Schisterman et al., 2009). Fourth, we performed multiple analyses in which we did not correct for multiple testing. Correction for multiple testing reduces the chance of type 1 error at the cost of increasing the risk for type 2 error. The markers for brain atrophy, CSVD, and cognitive impairment are correlated and based on the same shared risk factors and disease mechanisms. Furthermore, the magnitude of the associations is consistent and associations are directionally similar. Therefore, it is unlikely that our findings are a result of mere chance, against which multiple testing would safeguard. Fifth, we did not find an association between MDD and memory in our study sample with available MRI data, but the fact this association was present in the larger study sample including participants without MRI data suggests that depressed participants with MDD and poor memory were less likely to undergo MRI, leading to selection bias. This likely also applies to other population-imaging studies (Honningsvåg, Linde, Håberg, Stovner, & Hagen, 2012). Finally, we did not study segmented brain areas to assess region-specific differences such as hippocampal or prefrontal lobar volumes. Most evidence for brain atrophy in depression is found in focal volume loss rather than generalized volume loss (Sheline, 2000).

In a large cohort of community-dwelling participants aged between 40 and 75 years, MDD was associated with overall cognitive impairment and more impaired information processing speed and executive functioning & attention. MDD was further associated with CSVD in participants without T2DM, but this relation was insufficient to explain the associations between MDD and cognitive functioning. Longitudinal studies are needed to establish the mediation of structural brain damage in the development of cognitive impairment and dementia in MDD.

## Supplementary material

For supplementary material accompanying this paper visit https://doi.org/10.1017/S0033291721000222. click here to view supplementary material

## References

[ref1] Blazer, D. G. (2003). Depression in late life: Review and commentary. The Journals of Gerontology, 58, 249–265. doi: 10.1093/gerona/58.3.M249. 12634292

[ref2] Carney, R. M., Freedland, K. E., & Veith, R. C. (2005). Depression, the autonomic nervous system, and coronary heart disease. Psychosomatic Medicine, 67(Suppl 1), S29–S33. doi:10.1097/01.psy.0000162254.61556.d5. 15953797

[ref3] De Boer, R., Vrooman, H. A., Van Der Lijn, F., Vernooij, M. W., Ikram, M. A., Van Der Lugt, A., … Niessen, W. J. (2009). White matter lesion extension to automatic brain tissue segmentation on MRI. Neuroimage, 45, 1151–1161. doi:10.1016/j.neuroimage.2009.01.011. 19344687

[ref4] DeCarli, C., Fletcher, E., Ramey, V., Harvey, D., & Jagust, W. J. (2005). Anatomical mapping of white matter hyperintensities (WMH). Stroke, 36, 50–55. doi:10.1161/01.STR.0000150668.58689.f2. 15576652 PMC3816357

[ref5] Dotson, V. M., Davatzikos, C., Kraut, M. A., & Resnick, S. M. (2009). Depressive symptoms and brain volumes in older adults: A longitudinal magnetic resonance imaging study. Journal of Psychiatry and Neuroscience, 34, 367–375. 19721847 PMC2732743

[ref6] Fazekas, F., Chawluk, J. B., Alavi, A., Hurtig, H. I., & Zimmerman, R. A. (1987). MR Signal abnormalities at 1.5 T in Alzheimer's dementia and normal aging. AJR: American Journal of Roentgenology, 149, 351–356. doi:10.2214/ajr.149.2.351. 3496763

[ref7] Gregoire, S. M., Chaudhary, U. J., Brown, M. M., Yousry, T. A., Kallis, C., Jager, H. R., & Werring, D. J. (2009). The Microbleed Anatomical Rating Scale (MARS): Reliability of a tool to map brain microbleeds. Neurology, 73, 1759–1766. doi:10.1212/WNL.0b013e3181c34a7d. 19933977

[ref8] Hickie, I., Naismith, S., Ward, P. B., Turner, K., Scott, E., Mitchell, P., … Parker, G. (2005). Reduced hippocampal volumes and memory loss in patients with early-and late-onset depression. The British Journal of Psychiatry, 186, 197–202. doi:10.1192/bjp.186.3.197. 15738499

[ref9] Holt, R. I., De Groot, M., & Golden, S. H. (2014). Diabetes and depression. Current Diabetes Reports, 14, 491. doi:10.1007/s11892-014-0491-3.. 24743941 PMC4476048

[ref10] Honningsvåg, L.-M., Linde, M., Håberg, A., Stovner, L. J., & Hagen, K. (2012). Does health differ between participants and non-participants in the MRI-HUNT study, a population based neuroimaging study? The Nord-Trøndelag health studies 1984–2009. BMC Medical Imaging, 12. 10.1186/1471-2342-12-23.. PMC3472234 22846223

[ref11] Jernigan, T. L., Archibald, S. L., Fennema-Notestine, C., Gamst, A. C., Stout, J. C., Bonner, J., & Hesselink, J. R. (2001). Effects of age on tissues and regions of the cerebrum and cerebellum. Neurobiology of Aging, 22, 581–594. doi:10.1016/S0197-4580(01)00217-2. 11445259

[ref12] Koenig, A. M., Bhalla, R. K., & Butters, M. A. (2014). Cognitive functioning and late-life depression. Journal of the International Neuropsychological Society, 20, 461–467. doi:10.1017/S1355617714000198. 24685173 PMC4107679

[ref13] Köhler, S., Thomas, A. J., Barnett, N. A., & O'Brien, J. T. (2010). The pattern and course of cognitive impairment in late-life depression. Psychological Medicine, 40, 591–602. doi:10.1017/S0033291709990833. 19656429

[ref14] Köhler, S., Thomas, A. J., Lloyd, A., Barber, R., Almeida, O. P., & O'Brien, J. T. (2010). White matter hyperintensities, cortisol levels, brain atrophy and continuing cognitive deficits in late-life depression. The British Journal of Psychiatry, 196, 143–149. doi:10.1192/bjp.bp.109.071399. 20118461

[ref15] Li, W., Schram, M. T., Sorensen, B. M., Agtmaal, M. J. M., Berendschot, T., Webers, C. A. B., … Houben, A. (2020). Microvascular phenotyping in the Maastricht study: Design, and main findings, 2010–2018. American Journal of Epidemiology, 189, 873–884. 10.1093/aje/kwaa023. 32077474 PMC7443762

[ref16] Martens, R. J., Henry, R. M., Houben, A. J., van der Kallen, C. J., Kroon, A. A., Schalkwijk, C. G., … Stehouwer, C. D. (2016). Capillary rarefaction associates with albuminuria: The Maastricht study. Journal of the American Society of Nephrology, 27, 3748–3757. doi:10.1681/asn.2015111219. 27160406 PMC5118486

[ref17] Mourao, R. J., Mansur, G., Malloy-Diniz, L. F., Castro Costa, E., & Diniz, B. S. (2016). Depressive symptoms increase the risk of progression to dementia in subjects with mild cognitive impairment: Systematic review and meta-analysis. International Journal of Geriatric Psychiatry, 31, 905–911. doi:10.1002/gps.4406. 26680599

[ref18] Nouwen, A., Adriaanse, M., van Dam, K., Iversen, M., Viechtbauer, W., Peyrot, M., … de Groot, M. (2019). Longitudinal associations between depression and diabetes complications: A systematic review and meta-analysis. Diabetic Medicine, 36, 1562–1572. 10.1111/dme.14054.. 31215077

[ref19] O'Brien, J. T., Lloyd, A., McKeith, I., Gholkar, A., & Ferrier, N. (2004). A longitudinal study of hippocampal volume, cortisol levels, and cognition in older depressed subjects. American Journal of Psychiatry, 161, 2081–2090. doi:10.1176/appi.ajp.161.11.2081. 15514410

[ref20] O'Brien, J. T., & Thomas, A. J. (2015). Vascular dementia. The Lancet, 386, 1698–1706. doi:10.1016/S0140-6736(15)00463-8. 26595643

[ref21] Odaka, H., Adachi, N., & Numakawa, T. (2017). Impact of glucocorticoid on neurogenesis. Neural Regeneration Research, 12, 1028–1035. doi:10.4103/1673-5374.211174. 28852377 PMC5558474

[ref22] Pini, L., Pievani, M., Bocchetta, M., Altomare, D., Bosco, P., Cavedo, E., … Frisoni, G. B. (2016). Brain atrophy in Alzheimer's disease and aging. Ageing Research Reviews, 30, 25–48. doi:10.1016/j.arr.2016.01.002. 26827786

[ref23] Rayner, G., Jackson, G., & Wilson, S. (2016). Cognition-related brain networks underpin the symptoms of unipolar depression: Evidence from a systematic review. Neuroscience and Biobehavioral Reviews, 61, 53–65. doi: 10.1016/j.neubiorev.2015.09.022. 26562681

[ref24] Reijmer, Y. D., van den Berg, E., Ruis, C., Jaap Kappelle, L., & Biessels, G. J. (2010). Cognitive dysfunction in patients with type 2 diabetes. Diabetes/Metabolism Research and Reviews, 26, 507–519. doi: 10.1002/dmrr.1112. 20799243

[ref25] Rock, P., Roiser, J., Riedel, W., & Blackwell, A. (2014). Cognitive impairment in depression: A systematic review and meta-analysis. Psychological Medicine, 44, 2029–2040. doi:10.1017/S0033291713002535. 24168753

[ref26] Roy, T., & Lloyd, C. E. (2012). Epidemiology of depression and diabetes: A systematic review. Journal of Affective Disorders, 142, S8–S21. doi:10.1016/S0165-0327(12)70004-6. 23062861

[ref27] Ruigrok, A. N. V., Salimi-Khorshidi, G., Lai, M.-C., Baron-Cohen, S., Lombardo, M. V., Tait, R. J., & Suckling, J. (2014). A meta-analysis of sex differences in human brain structure. Neuroscience and Biobehavioral Reviews, 39, 34–50. doi:10.1016/j.neubiorev.2013.12.004. 24374381 PMC3969295

[ref28] Schisterman, E. F., Cole, S. R., & Platt, R. W. (2009). Overadjustment bias and unnecessary adjustment in epidemiologic studies. Epidemiology (Cambridge, Mass.), 20, 488. doi: 10.1097/EDE.0b013e3181a819a1. 19525685 PMC2744485

[ref29] Schram, M. T., Sep, S. J., van der Kallen, C. J., Dagnelie, P. C., Koster, A., Schaper, N., … Stehouwer, C. D. (2014). The Maastricht study: An extensive phenotyping study on determinants of type 2 diabetes, its complications and its comorbidities. European Journal of Epidemiology, 29, 439–451. doi:10.1007/s10654-014-9889-0. 24756374

[ref30] Sheehan, D. V., Lecrubier, L., Sheehan, K. H., Amorim, P., Janavs, J., Weiller, E., Dunbar, G. C., … Dunbar, G. C. (1998). The Mini-international neuropsychiatric interview (MINI): The development and validation of a structured diagnostic psychiatric interview for DSM-IV and ICD-10. Journal of Clinical Psychiatry, 59, 22–33. 9881538

[ref31] Sheline, Y. I. (2000). 3D MRI studies of neuroanatomic changes in unipolar major depression: The role of stress and medical comorbidity. Biological Psychiatry, 48, 791–800. doi:10.1016/S0006-3223(00)00994-X. 11063975

[ref32] Sheline, Y. I., Price, J. L., Vaishnavi, S. N., Mintun, M. A., Barch, D. M., Epstein, A. A., … Schechtman, K. (2008). Regional white matter hyperintensity burden in automated segmentation distinguishes late-life depressed subjects from comparison subjects matched for vascular risk factors. American Journal of Psychiatry, 165, 524–532. doi:10.1176/appi.ajp.2007.07010175. 18281408 PMC4118770

[ref33] Sneed, J. R., Rindskopf, D., Steffens, D. C., Krishnan, K. R. R., & Roose, S. P. (2008). The vascular depression subtype: Evidence of internal validity. Biological Psychiatry, 64, 491–497. doi: 10.1016/j.biopsych.2008.03.032. 18490003 PMC2597784

[ref34] Thomas, A. J., & O'Brien, J. T. (2008). Depression and cognition in older adults. Current Opinion in Psychiatry, 21, 8–13. doi:10.1097/YCO.0b013e3282f2139b. 18281834

[ref35] Thompson, P. M., Hayashi, K. M., de Zubicaray, G., Janke, A. L., Rose, S. E., Semple, J., … Toga, A. W. (2003). Dynamics of gray matter loss in Alzheimer's disease. Journal of Neuroscience, 23, 994–1005. doi: 10.1523/JNEUROSCI.23-03-00994.2003. 12574429 PMC6741905

[ref36] Tittmann, M., Günther, T., Sacher, J., Himmerich, H., Villringer, A., Hegerl, U., & Schönknecht, P. (2014). Structural brain changes in early-onset and late-onset depression: An update of volumetric MRI findings. International Journal of Imaging Systems and Technology, 24, 149–160. doi:10.1002/ima.22089.

[ref37] Tudorascu, D. L., Rosano, C., Venkatraman, V. K., MacCloud, R. L., Harris, T., Yaffe, K., … Aizenstein, H. J. (2014). Multimodal MRI markers support a model of small vessel ischemia for depressive symptoms in very old adults. Psychiatry Research, 224, 73–80. doi:10.1016/j.pscychresns.2014.08.009. 25205441 PMC4195799

[ref38] van Agtmaal, M. J., Houben, A. J., de Wit, V., Henry, R. M., Schaper, N. C., Dagnelie, P. C., … Kroon, A. A. (2018). Prediabetes is associated with structural brain abnormalities: The Maastricht study. Diabetes Care, 41, 2535–2543. doi:10.2337/dc18-1132. 30327356

[ref39] van Agtmaal, M. J., Houben, A. J., Pouwer, F., Stehouwer, C. D., & Schram, M. T. (2017). Association of microvascular dysfunction with late-life depression: A systematic review and meta-analysis. JAMA Psychiatry, 74, 729–739. doi:10.1001/jamapsychiatry.2017.0984. 28564681 PMC5710252

[ref40] Van Der Elst, W., Van Boxtel, M. P., Van Breukelen, G. J., & Jolles, J. (2005). Rey's verbal learning test: Normative data for 1855 healthy participants aged 24–81 years and the influence of age, sex, education, and mode of presentation. Journal of the International Neuropsychological Society, 11, 290–302. doi: 10.1017/S1355617705050344. 15892905

[ref41] Van der Elst, W., Van Boxtel, M. P., Van Breukelen, G. J., & Jolles, J. (2006a). The concept shifting test: Adult normative data. Psychological Assessment, 18, 424. doi:10.1037/1040-3590.18.4.424. 17154763

[ref42] Van der Elst, W., van Boxtel, M. P., van Breukelen, G. J., & Jolles, J. (2006b). The letter digit substitution test: Normative data for 1858 healthy participants aged 24–81 from the Maastricht Aging Study (MAAS): Influence of age, education, and sex. Journal of Clinical and Experimental Neuropsychology, 28, 998–1009. doi:10.1080/13803390591004428. 16822738

[ref43] Van der Elst, W., Van Boxtel, M. P., Van Breukelen, G. J., & Jolles, J. (2006c). The stroop color-word test: Influence of age, sex, and education; and normative data for a large sample across the adult age range. Assessment, 13, 62–79. doi:10.1177/1073191105283427. 16443719

[ref44] Vergoossen, L. W., Jansen, J. F., Backes, W. H., & Schram, M. T. (2020). Cardiometabolic determinants of early and advanced brain alterations: Insights from conventional and novel MRI techniques. Neuroscience and Biobehavioral Reviews, 115, 308–320. 10.1016/j.neubiorev.2020.04.001.. 32439370

[ref45] Vrooman, H. A., Cocosco, C. A., van der Lijn, F., Stokking, R., Ikram, M. A., Vernooij, M. W., … Niessen, W. J. (2007). Multi-spectral brain tissue segmentation using automatically trained k-nearest-neighbor classification. Neuroimage, 37, 71–81. doi:10.1016/j.neuroimage.2007.05.018. 17572111

[ref46] Wardlaw, J. M., Smith, E. E., Biessels, G. J., Cordonnier, C., Fazekas, F., Frayne, R., … Dichgans, M. (2013). Neuroimaging standards for research into small vessel disease and its contribution to ageing and neurodegeneration. Lancet Neurology, 12, 822–838. doi:10.1016/s1474-4422(13)70124-8. 23867200 PMC3714437

[ref47] WHO (2006). Definition and diagnosis of diabetes mellitus and intermediate hyperglycaemia: Report of a WHO/IDF consultation. Geneva, Switzerland: World Health Organization.

[ref48] WHO (2017). Depression and other common mental disorders: Global health estimates. Geneva, Switzerland: World Health Organization.

