# Supplementary material for: The relation of depression with structural brain abnormalities and cognitive functioning: the Maastricht study
Source: Psychol Med. 2021 Feb 26;52(15):3521–30. doi: 10.1017/S0033291721000222 (PMC9772903; doi:10.1017/S0033291721000222)
Supplement: Supplementary file 1 [file S0033291721000222sup001.docx]

**Supplemental Material**

**The relation of depression with structural brain abnormalities and cognitive functioning: The Maastricht Study**

Anouk FJ Geraets^1,2,3,7,8^, Miranda T Schram^2,3,4,7,8^, Jacobus FA Jansen^5,7^, Annemarie Koster^6,9^, Pieter C Dagnelie^3,8^, Marleen MJ van Greevenbroek^3,8^, Coen DA Stehouwer^3,8^, Frans RJ Verhey^1,2,7^, & Sebastian Köhler^1,2,7^

^1^Alzheimer Centrum Limburg, ^2^Department of Psychiatry and Neuropsychology, ^3^Department of Internal Medicine, ^4^Heart and Vascular Centre, ^5^Department of Radiology, ^6^Department of Social Medicine, Maastricht University Medical Centre (MUMC+), Maastricht, the Netherlands; ^7^School for Mental Health and Neuroscience, ^8^School for Cardiovascular Diseases (CARIM), ^9^Care and Public Health Research Institute (CAPHRI), Faculty of Health, Medicine & Life Sciences, Maastricht University, Maastricht, the Netherlands

**Table S1**. Interaction analyses of associations of major depressive disorder with different domains of cognitive functioning

|  | **Memory score** |  | **Information processing speed score** |  | **Executive functioning & attention score** |  | **Cognitive impairment (yes/no)** |  |
| --- | --- | --- | --- | --- | --- | --- | --- | --- |
| **Model 3** | **Mean difference (95% CI)** | ***P-*value** | **Mean difference (95% CI)** | ***P-*value** | **Mean difference (95% CI)** | ***P-*value** | **OR**  **(95% CI)** | ***P-*value** |
| **MDD*age** | -0.01(-0.21;0.11) | 0.526 | -0.01(-0.02;0.00) | 0.187 | 0.01(-0.01;0.02) | 0.448 | 1.00(0.96;1.05) | 0.955 |
| **MDD*women** | -0.04(-0.30;0.22) | 0.762 | 0.01(-0.20;0.21) | 0.934 | 0.01(-0.22;0.24) | 0.943 | 0.67(0.30;1.50) | 0.330 |
| **MDD*T2DM** | 0.08(-0.20;0.36) | 0.563 | -0.03(-0.25;0.19) | 0.762 | -0.07(-0.31;0.18) | 0.586 | 1.66(0.74;3.72) | 0.216 |

n=4,734. Major depressive disorder cases n=151. Regression results are presented as mean difference or odds ratio (OR) with 95% confidence intervals (95% CI). CI indicates confidence interval; SD, standard deviation; OR, odds ratio; MDD, major depressive; T2DM, type 2 diabetes mellitus.

**Model 3:** adjusted for intracranial volume (except CSVD composite score), MRI lag time, age, sex, educational level, and type 2 diabetes mellitus.

**Table S2**. Interaction analyses of associations of major depressive disorder with markers of brain atrophy

|  | CSF volume  (per 1 SD) |  | WM volume  (per 1 SD) |  | GM volume  (per 1 SD) |  |
| --- | --- | --- | --- | --- | --- | --- |
| Model 3 | **Mean difference (95% CI)** | ***P-*value** | **Mean difference (95% CI)** | ***P-*value** | **Mean difference (95% CI)** | ***P-*value** |
| MDD*age | 0.00(-0.01;0.01) | 0.776 | 0.01(0.00;0.02) | 0.029 | -0.01(-0.02;-0.00) | 0.013 |
| MDD*women | 0.10(-0.10;0.31) | 0.334 | -0.08(-0.23;0.07) | 0.290 | -0.00(-0.15;0.14) | 0.961 |
| MDD*T2DM | -0.09(-0.32;0.13) | 0.402 | -0.03(-0.19;0.13) | 0.689 | 0.12(-0.04;0.27) | 0.142 |

n=4,734. Major depressive disorder cases n=151. Regression results are presented as mean difference or odds ratio (OR) with 95% confidence intervals (95% CI). CSF indicates cerebrospinal fluid; WM, white matter; GM, grey matter; SD, standard deviation; MDD, major depressive disorder; T2DM, type 2 diabetes mellitus.

**Model 3:** adjusted for intracranial volume (except CSVD composite score), MRI lag time, age, sex, educational level, and type 2 diabetes mellitus.

**Table S3**. Interaction analyses of associations of major depressive disorder with markers of cerebral small vessel disease

|  | WMH volume (per 1 SD) |  | DWMH volume (per 1 SD) |  | PWMH volume (per 1 SD) |  | CSVD  (yes/no) |  |
| --- | --- | --- | --- | --- | --- | --- | --- | --- |
| Model 3 | **Mean difference**  **(95% CI)** | ***P-*value** | **Mean difference (95% CI)** | ***P-*value** | **Mean difference (95% CI)** | ***P-*value** | **OR**  **(95% CI)** | ***P-*value** |
| MDD*age | 0.01(-0.01;0.03) | 0.330 | 0.01(-0.01;0.03) | 0.330 | 0.14(-0.00;0.03) | 0.110 | 0.99(0.95;1.04) | 0.774 |
| MDD*women | -0.13(-0.41;0.16) | 0.390 | -0.13(-0.41;0.16) | 0.390 | 0.02(-0.26;0.30) | 0.874 | 1.23(0.59;2.55) | 0.583 |
| MDD*T2DM | -0.14(-0.44;0.16) | 0.355 | -0.21(-0.52;0.10) | 0.186 | -0.12(-0.42;0.18) | 0.422 | 0.40(0.18;0.90) | 0.026 |

n=4,734. Major depressive disorder cases n=151. Regression results are presented as mean difference or odds ratio (OR) with 95% confidence intervals (95% CI). WMH indicates white matter hyperintensity; DWMH, deep cortical white matter hyperintensities; PWMH, periventricular white matter hyperintensities; CSVD, cerebral small vessel disease; SD, standard deviation; MDD, major depressive disorder; T2DM, type 2 diabetes mellitus.

**Model 3:** adjusted for intracranial volume (except CSVD composite score), MRI lag time, age, sex, educational level, and type 2 diabetes mellitus.

**Table S4**. Decomposed associations of major depressive disorder with different domains of cognitive functioning in the total study population

|  | **Memory score** | **Information processing speed score** | **Executive functioning & attention score** | **Cognitive impairment (yes/no)** |
| --- | --- | --- | --- | --- |
| **Model** | **Mean difference (95% CI)** | **Mean difference (95% CI)** | **Mean difference (95% CI)** | **OR (95% CI)** |
| **WMH** |  |  |  |  |
| Direct | -0.07(-0.20;0.06) | -0.18(-0.28;-0.08)* | -0.14(-0.26;0.03)* | 1.35(1.07;1.70)* |
| Indirect | -0.01(-0.02;0.00) | -0.01(-0.01;0.00) | -0.01(-0.01;0.00) | 1.02(1.00;1.03) |
| Total | -0.08(-0.21;0.05) | -0.19(-0.29;-0.09)* | -0.15(-0.26;-0.03)* | 1.37(1.09;1.73)* |
| **DWMH** |  |  |  |  |
| Direct | -0.07(-0.20;0.06) | -0.19(-0.29;-0.08)* | -0.14(-0.26;-0.03)* | 1.35(1.07;1.71)* |
| Indirect | -0.01(-0.02;0.00) | -0.00(-0.01;0.00) | -0.00(-0.01;0.00) | 1.01(0.99;1.03) |
| Total | -0.08(-0.21;0.05) | -0.19(-0.29;-0.09)* | -0.15(-0.26;-0.03)* | 1.37(1.08;1.73)* |
| **PWMH** |  |  |  |  |
| Direct | -0.07(-0.20;0.06) | -0.18(-0.29;-0.08)* | -0.14(-0.25;-0.03)* | 1.35(1.07;1.70)* |
| Indirect | -0.01(-0.02;0.00) | -0.01(-0.02;0.00) | -0.01(-0.02;0.00) | 1.01(1.00;1.03) |
| Total | -0.08(-0.21;0.05) | -0.19(-0.29;-0.09)* | -0.15(-0.26;-0.03)* | 1.36(1.08;1.72)* |
| **CSVD** |  |  |  |  |
| Direct | -0.06(-0.20;0.08) | -0.21(-0.31;-0.12)* | -0.20(-0.30;-0.10)* | 1.37(1.09;1.73)* |
| Indirect | -0.00(-0.01;0.00) | -0.00(-0.01;0.00) | -0.00(-0.01;0.00) | 1.01(0.99;1.03) |
| Total | -0.07(-0.21;0.07) | -0.22(-0.32;-0.12)* | -0.20(-0.30;-0.11)* | 1.39(1.10;1.75)* |

Regression results are decomposed in direct, indirect and total effects and presented as mean difference or odds ratio (OR) with 95% confidence intervals (95% CI). CI indicates confidence interval; OR, odds ratio; MDD, major depressive; WMH, white matter hyperintensities; DWMH, deep cortical white matter hyperintensities; PWMH, periventricular white matter hyperintensities; CSVD, cerebral small vessel disease. Model 2 is adjusted for adjusted for age, sex, educational level. n=4,734, MDD cases n=151. **p* < 0.05 (two-sided).

**Table S5**. Associations of major depressive disorder with different domains of cognitive functioning in participants with and without missing MRI data

|  | **Memory score** | | |  | **Information processing speed score** |  | **Executive functioning & attention score** | |  | **Cognitive impairment (yes/no)** |  |  |
| --- | --- | --- | --- | --- | --- | --- | --- | --- | --- | --- | --- | --- |
| **Model** | **Mean difference (95% CI)** | | | ***P-*value** | **Mean difference**  **(95% CI)** | ***P-*value** | **Mean difference**  **(95% CI)** | ***P-*value** | | **OR**  **(95% CI)** | ***P-*value** |  |
| **Study population with MRI data^a^** | |  | |  |  |  |  |  | |  |  | |
| Model 1 | -0.15(-0.30;0.01) | | | 0.061 | -0.27(-0.40;-0.15) | <0.001 | -0.23(-0.36;-0.11) | <0.001 | | 1.83(1.24;2.70) | 0.002 |  |
| Model 2 | -0.08(-0.21;0.06) | | | 0.257 | -0.22(-0.33;-0.12) | <0.001 | -0.18(-0.29;-0.06) | 0.002 | | 1.89(1.28;2.79) | 0.001 |  |
| Model 3 | -0.06(-0.19;0.07) | | | 0.373 | -0.21(-0.31;-0.10) | <0.001 | -0.17(-0.28;-0.05) | 0.005 | | 1.79(1.21;2.66) | 0.004 |  |
| Model 4 | -0.05(-0.18;0.08) | | | 0.447 | -0.20(-0.30;-0.10) | <0.001 | -0.16(-0.27;-0.04) | 0.008 | | 1.74(1.17;2.59) | 0.007 |  |
| Model 5 | -0.02(-0.15;0.11) | | | 0.735 | -0.18(-0.28;-0.08) | 0.001 | -0.13(-0.25;-0.02) | 0.026 | | 1.60(1.06;2.40) | 0.024 |  |
| **Study population with missing MRI data**^b^ | | |  |  |  |  |  |  | |  |  | |
| Model 1 | -0.25(-0.37;-0.13) | | | <0.001 | -0.35(-0.45;-0.25) | <0.001 | -0.35(-0.45;-0.24) | <0.001 | | 2.73(2.07;3.61) | <0.001 |  |
| Model 2 | -0.18(-0.28;-0.07) | | | 0.001 | -0.29(-0.38;-0.21) | <0.001 | -0.28(-0.38;-0.19) | <0.001 | | 2.80(2.11;3.70) | <0.001 |  |
| Model 3 | -0.16(-0.26;-0.05) | | | 0.003 | -0.27(-0.35;-0.19) | <0.001 | -0.26(-0.36;-0.17) | <0.001 | | 2.64(1.99;3.50) | <0.001 |  |
| Model 4 | -0.14(-0.25;-0.04) | | | 0.008 | -0.26(-0.35;-0.18) | <0.001 | -0.26(-0.35;-0.16) | <0.001 | | 2.57(1.93;3.43) | <0.001 |  |
| Model 5 | -0.11(-0.22;-0.00) | | | 0.044 | -0.24(-0.32;-0.15) | <0.001 | -0.23(-0.32;-0.14) | <0.001 | | 2.37(1.77;3.17) | <0.001 |  |

^a^n=4,734. Major depressive disorder cases n=151.

^b^n=7,066. Major depressive disorder cases n=236.

Regression results are presented as mean difference or odds ratio (OR) with 95% confidence intervals (95% CI). CI indicates confidence interval; SD, standard deviation; OR, odds ratio.

**Model 1:** crude.

**Model 2:** adjusted for age, sex, and educational level.

**Model 3:** additionally adjusted for type 2 diabetes mellitus.

**Model 4:** additionally adjusted for waist circumference, office systolic blood pressure, hypertensive medication, total/high density cholesterol ratio, lipid modifying medication, and history of cardiovascular disease (n=4,701 and n=7,006 in study population without and with missing MRI data respectively).

**Model 5:** additionally adjusted for smoking behaviour and alcohol use (n=4.698 and n=6,998 in study population without and with missing MRI data respectively).

**Table S6**. Sensitivity analyses of associations of major depressive disorder with different domains of cognitive functioning

|  | **Memory score** |  | **Information processing speed score** |  | **Executive functioning & attention score** |  | **Cognitive impairment (yes/no)** |  |
| --- | --- | --- | --- | --- | --- | --- | --- | --- |
| **Model** | **Mean difference (95% CI)** | ***P-*value** | **Mean difference (95% CI)** | ***P-*value** | **Mean difference (95% CI)** | ***P-*value** | **OR**  **(95% CI)** | ***P-*value** |
| Model 5 | -0.02(-0.15;0.11) | 0.735 | -0.18(-0.28;-0.08) | 0.001 | -0.13(-0.25;-0.02) | 0.026 | 1.60(1.06;2.40) | 0.024 |
| Model 6: model 5 + antidepressant medication | 0.00(-0.13;0.13) | 0.994 | -0.16(-0.26;-0.05) | 0.003 | -0.10(-0.22;0.01) | 0.083 | 1.50(1.00;2.27) | 0.053 |
| Model 7: model 5 excl. antidepressant users from controls | -0.02(-0.15;0.11) | 0.735 | -0.18(-0.28;-0.08) | 0.001 | -0.13(-0.25;-0.02) | 0.026 | 1.60(1.06;2.40) | 0.024 |
| Model 8: model 5 excl. PHQ-9 score ≥10 from control | -0.02(-0.15;0.11) | 0.735 | -0.18(-0.28;-0.08) | 0.001 | -0.13(-0.25;-0.02) | 0.026 | 1.86(1.05;3.31) | 0.035 |
| Model 9: model 5 excl. lifetime MDD from controls | -0.02(-0.15;0.11) | 0.735 | -0.18(-0.28;-0.08) | 0.001 | -0.13(-0.25;-0.02) | 0.026 | 1.86(1.05;3.31) | 0.035 |
| Model 10: model 5 + physical activity^a^ | -0.05(-0.18;0.10) | 0.529 | -0.21(-0.32;-0.10) | <0.001 | -0.15(-0.27;-0.02) | 0.019 | 1.62(1.04;2.53) | 0.034 |
| Model 11: model 5 + Mediterranean diet score^b^ | -0.03(-0.16;0.11) | 0.690 | -0.21(-0.32;-0.10) | 0.001 | -0.13(-0.25;-0.01) | 0.031 | 1.66(1.09;2.53) | 0.018 |
| Model 12: model 5 + eGFR^c^ | -0.03(-0.22;0.17) | 0.798 | -0.13(-0.29;0.02) | 0.093 | -0.18(-0.35;-0.01) | 0.037 | 2.07(1.18;3.66) | 0.012 |
| Model 13: model 5 replacing office SBP for 24h SBP^d^ | -0.08(-0.30;0.14) | 0.485 | -0.12(-0.29;0.05) | 0.158 | -0.15(-0.34;0.04) | 0.127 | 1.57(0.80;3.07) | 0.191 |
| Model 14: model 5 replacing waist circumference for BMI | -0.02(-0.15;0.11) | 0.734 | -0.18(-0.28;-0.07) | 0.001 | -0.13(-0.25;-0.01) | 0.027 | 1.59(1.06;2.39) | 0.025 |
| Model 15: model 5 replacing total-to-HDL cholesterol ratio for triglycerides | -0.02(-0.15;0.11) | 0.742 | -0.18(-0.28;-0.08) | 0.001 | -0.13(-0.25;-0.02) | 0.025 | 1.60(1.07;2.40) | 0.023 |

Regression results are presented as mean difference or odds ratio (OR) with 95% confidence intervals (95% CI). CI indicates confidence interval; SD, standard deviation; OR, odds ratio; MDD, major depressive; eGFR, estimated glomerular filtration rate; SBP, systolic blood pressure. Model 5 (n=4,734, MDD cases n=151) is adjusted for adjusted for age, sex, educational level, type 2 diabetes mellitus, waist circumference, office systolic blood pressure, hypertensive medication, total/high density cholesterol ratio, lipid modifying medication, history of cardiovascular disease, smoking behavior and alcohol use. ^a^n=4,249, ^b^n=4,459, ^c^n=2,024, ^d^n=1,805

**Table S7**. Age trend for the associations of major depressive disorder with markers of brain atrophy and cerebral small vessel disease

|  | CSF volume  (per 1 SD) | WM volume  (per 1 SD) | GM volume  (per 1 SD) | WMH volume (per 1 SD) | DWMH volume (per 1 SD) | PWMH volume (per 1 SD) | CSVD  (yes/no) |
| --- | --- | --- | --- | --- | --- | --- | --- |
| Model 3 | **Mean difference**  **(95% CI)** | **Mean difference**  **(95% CI)** | **Mean difference**  **(95% CI)** | **Mean difference**  **(95% CI)** | **Mean difference (95% CI)** | **Mean difference (95% CI)** | **OR**  **(95% CI)** |
| Aged 40-50 years | -0.09(-0.31;0.13) | -0.06(-0.23;0.11) | 0.13(-0.02;0.29) | -0.22(-0.51;0.06) | 0.01(-0.24;0.26) | -0.29(0.57;0.00) | 2.45(0.94;6.42) |
| Aged 50-60 years | -0.02(-0.17;0.14) | -0.03(-0.14;0.09) | 0.04(-0.07;0.15) | 0.20(-0.01;0.42) | -0.02(-0.23;0.19) | 0.27(0.06;0.48) | 0.85(0.46;1.57) |
| Aged 60-70 years | 0.09(-0.10;0.28) | 0.01(-0.13;0.15) | -0.08(-0.22;0.06) | 0.25(-0.02;0.52) | 0.25(-0.04;0.54) | 0.25(-0.02;0.51) | 1.44(0.79;2.63) |
| Aged 70-80 years | -0.03(-0.40;0.35) | 0.23(-0.02;0.48) | -0.20(-0.47;0.07) | 0.18(-0.30;0.65) | 0.26(-0.32;0.83) | 0.17(-0.30;0.63) | 1.16(0.39;3.46) |

^a^n=891. Major depressive disorder cases n=28.

^b^n=1,573. Major depressive disorder cases n=63.

^c^n=1,841. Major depressive disorder cases n=46.

^d^n=429. Major depressive disorder cases n=14.

Regression results are presented as mean difference or odds ratio (OR) with 95% confidence intervals (95% CI). CSF indicates cerebrospinal fluid; WM, white matter; GM, grey matter; SD, standard deviation; WMH, white matter hyperintensity; DWMH, deep cortical white matter hyperintensities; PWMH, periventricular white matter hyperintensities; CSVD, cerebral small vessel disease; SD, standard deviation.

**Model 3:** adjusted for intracranial volume (except CSVD composite score), MRI lag time, age, sex, educational level, and type 2 diabetes mellitus.
